# Incidence of the thermal transition in the range of 45-5°C in chromatophores present in the wings of *Schistocerca americana*

**DOI:** 10.12688/f1000research.18314.1

**Published:** 2019-02-27

**Authors:** María Belén Cañizares, Nathaly Naranjo, Bence Mátyás

**Affiliations:** 1 Universidad Central del Ecuador, Cayambe, Ecuador; 2 Grupo de Investigación en Ciencias Ambientales (GRICAM), Universidad Politécnica Salesiana, Quito, Ecuador

**Keywords:** Schistocerca americana, Thermal transition, Chromatophores, Melanism, Pigmentary coloration

## Abstract

The variation of the color intensity of the chromatophores present in the wings of *Schistocerca americana* was analyzed by exposing 31 specimens to thermal transitions within the range of 45 - 5 °C.  The adult specimens were collected using a mini-terrarium of dimensions 40x40x30 cm. As a substrate, a layer of soil, stones, and finally a layer of grass were used along with branches of bushes and leaves; hydroponic lettuce, cabbage and the grass were used as food for the specimens. Optical microscopy of the wings of the insects was used for live observation without coverslips or contrasting substances. At 45°C, degradation of color intensity was observed in the chromatophores present in the wings. At 5°C, chromatophores intensify their color to brownish-black. This temperature was the extreme minimum that *S. americana* could tolerate. We found negative correlation between the temperature and the degree of darkness (R2 = 0.8038). Our results are in accordance with a previously published study in which *Phaulacridium vittatum* was examined, as the decrease of temperature caused darkening color change in melanin-type chromatophores. The present investigation can be considered as the first initial study of its kind for *S. americana*, in terms of examining the changes in the color intensity of the chromatophores present in the wings caused by thermal transition under laboratory conditions.

## Introduction

Melanism, the occurrence of darker pigmentation in specimens within species is well-known in insects ^1, 2^. It is also known that chromatophores to those cells present in the animal integumentary system containing pigments, and these cells respond to hormonal factors and/or neuronal (response to stimuli) factors ^2^. There are several studies focusing on why melanism has evolved, and a hypothesis about this is known as thermal melanism hypothesis ^2, 3^. This states that at low temperatures, darker insects are at an advantage compared to light ones because they warm up more quickly. There are several studies that support the thermal melanism hypothesis related to butterflies ^3– 5^ and hoverflies ^6^.

Insects have a chemical or pigmentary coloration represented mainly by carotene and melanin, which are found in the cuticle, hypodermis or sub-hypodermis ^7^. In the exocuticle, it is more common to find the variants of carotene and melanin as yellow, brown and black colorings, and in the hypodermis as yellow, red, green and orange colorings ^8^. Adult *Schistocerca americana* have wings with large brown spots (melanin) on a light-colored background ^9^. The study of the Arthropoda phylum and its Insecta class is highly wide-spread, however, the majority of the studies regarding grasshoppers' behavior under different temperature conditions, focus on habitat selection only ^10, 11^.

The main objective of this study was to analyze the effect of thermal transition on the intensity of the color of the chromatophores present in the wings of the species *S. Americana.* This study was inspired by the observation in the literature ^3– 6^ that temperature has a direct effect on change and/or color variations of the chromatophores located in other species and phyla of the Animalia kingdom.

## Methods

### Specimen collection

A total of 31 adult *S. americana* were collected by hand according to Harris *et al.* 2013 ^3^ in Tumbaco- Quito Ecuador ** (GPS coordinates: 0°13'19.1"S 78°22'18.2"W) in May, 2017 *.* No permits were needed for collecting the specimens considering that the S. *americana* does not appear in the red list ( IUCN), and the sampling site is not located in a protected area. Nevertheless, prior consultation was carried out with the local authorities (Mayor's office and Ministry of Environment of Ecuador). All efforts were made to ameliorate any suffering to the animals following the protocol described by the Ecuadorian code of practice for the care and use of animals for scientific purposes of the Ministry of Environment of Ecuador.

### Housing of specimens

**Figure 1.**
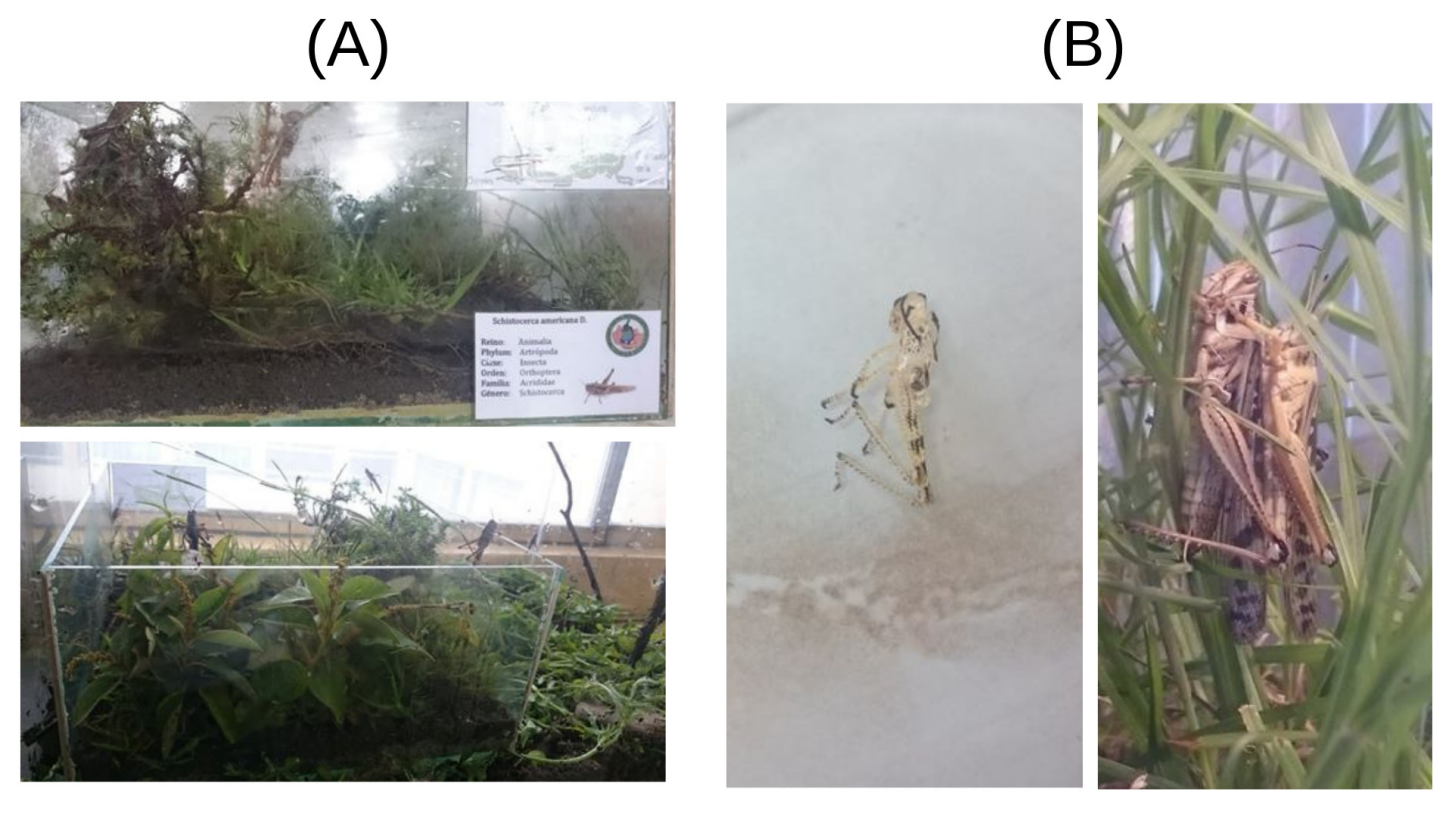
Terrariums used in the study and evidence of adaption. ( **A** top) Mini terrarium for collection; ( **A** bottom) habitat for the specimens during the experiment; ( **B** left) Evidence of adaptation, molt; ( **B** right) Evidence of adaptation, reproduction.

Three terrariums were used in the experiment: one with the dimensions 40x40x30 cm and two with the dimensions 70x70x50 cm ( Figure 1A). The first terrarium was used for the collection of specimens in the and the others of greater longevity for the creation of an adaptation habitat and for experimentation, respectively. As a substrate, a layer of soil (approx. 4cm) collected in the same location, one layer of stones (approx. 2 cm), and finally a layer of grass (approx. 2cm) was used along with branches of bushes and leaves ( Figure 1A). A plastic mesh was used to cover the terrariums. Hydroponic lettuce, cabbage and the grass were used as food for the specimens.

### Thermal transition

To ensure adaption from nature to laboratory conditions, 25°C was applied for 7 days before the heat treatments. To observe the color changes, in the laboratory, the specimens were exposed to temperatures of: 45°C, 40°C, 35°C, 30°C, 25°C and 5°C for which a system of five 100w lightbulbs were used as a source of heat. Every two days a light bulb was switched off in order to ensure the decrease of temperature. For low temperature (5°C) all the lights were turned off in the system and dry ice was placed directly on the substrate.

### Data collection

For the microscopic observation, the specimens were sacrificed at -4℃, and the primary wings were removed from the insects using entomological tweezers, avoiding tearing them, at room temperature according to the method by Paredes *et al*. ^12^. Optical microscopy (Carl Zeiss, model: Laboval 4) was used for live observation of the color change without coverslip or contrast.

For color interpretation, we used hexadecimal classification by selecting the wing spot areas using iVinci Express v.4.6 on a 32 scale where the degree of darkness is interpreted in percentage using 0to255 software tool (see the color codes used in the analysis in *Underlying data* ^13^). Linear regression was applied for analyzing the correlation between the temperature and the degree of darkness in Microsoft Office 365.

## Results and Discussions

Adaption to the terrarium environment was considered successful as after a one-week period the specimens began molting ( Figure 1B left) and reproducing ( Figure 1B right).

**Figure 2.**
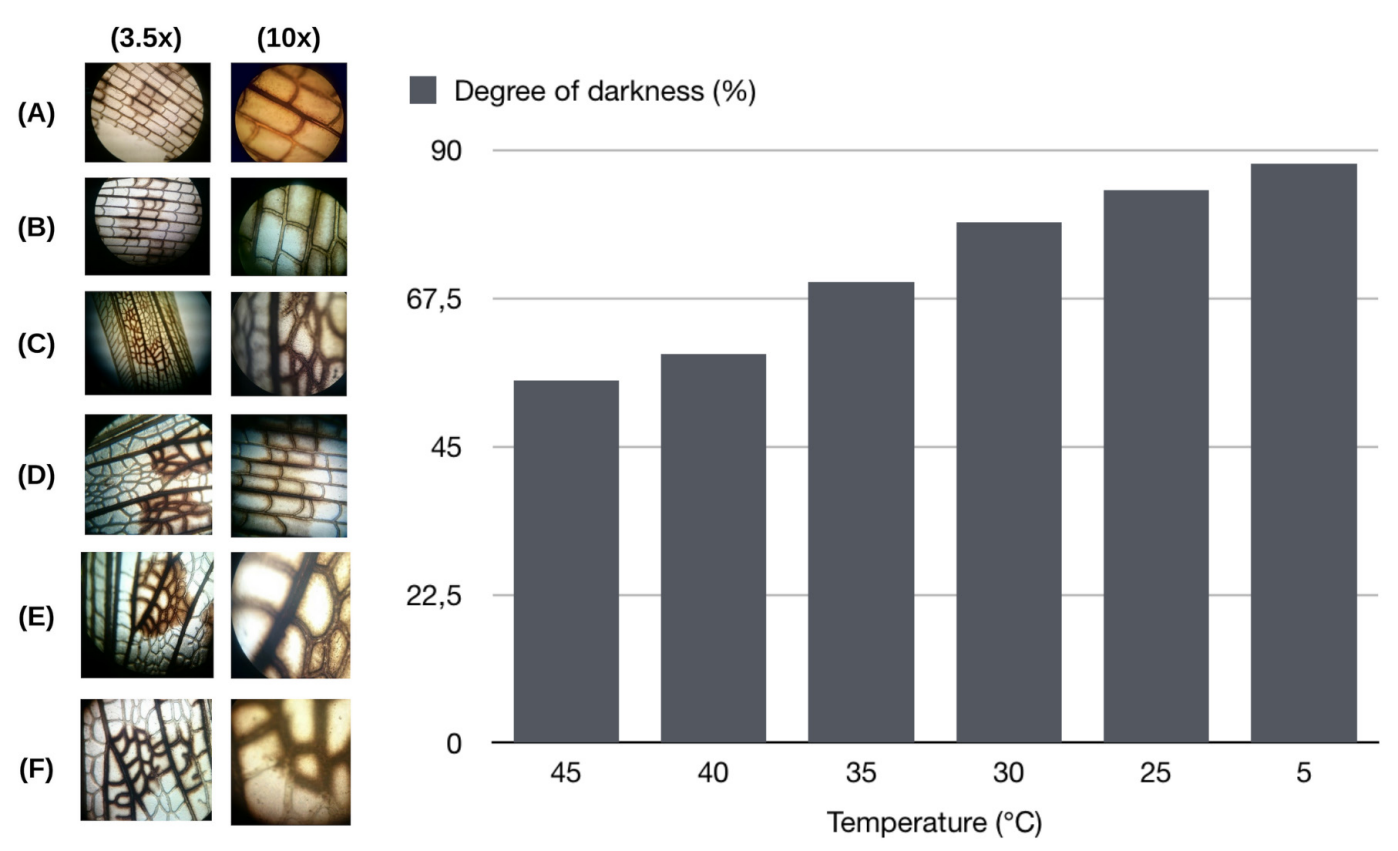
Results of the incidence of the thermal transition in the range of 45–5°C in chromatophores present in the wings of *Schistocerca americana*. Chromatophores (melanin) at a temperature of ( **A**) 45 ^⍜^C; ( **B**) 40 ^⍜^C; ( **C**) 35 ^⍜^C; ( **D**) 30 ^⍜^C; ( **E**) 25 ^⍜^C; ( **F**) 5 ^⍜^C. Magnifications: 3.5 x (left), and 10x (right).

At 45°C, degradation of color intensity can be observed in chromatophores present in the wings ( Figure 2A). At 40°C chromatophores of the melanin-type begin to darken ( Figure 2B); however, between the temperature of 45°C and 40°C there are no huge differences in color change. At 35°C chromatophores of the wings take a light brown color, intensify their color ( Figure 2C). At 30°C the chromatophores turn dark brown, indicating an increase in their color intensity ( Figure 2D). A temperature of 25°C corresponds to the optimum temperature for the normal development of the species in the region ^14^ ( Figure 2E). This is the reference color of the chromatophores used for all analysis. At 5°C chromatophores of the melanin-type intensify their color to brownish-black ( Figure 2F). This temperature was the extreme minimum that *S. americana* could tolerate.

Temperature, precipitation and solar radiation are the meteorological elements that most affect the distribution, rate of growth, reproduction, migration and adaptation of insects ^15^. The thermal melanism in tests with *Phaulacridium vittatum* and its exposure to the heat of lights within the range 300 to 700 nm represented an intensification in its color with a variation in percentage of between 2.49% to 5.65% ^3^. The majority of the studies that examine the thermal melanism have focused on species with very distinct color morphs representing a wide range in melanism.

We found negative correlation between the temperature and the degree of darkness (R2 = 0.8038). Our results are in accordance with a previously published study ^3^ in which *P. vittatum* was examined - a decrease of temperature caused darkening color change in melanin-type chromatophores.

## Conclusions

The present investigation can be considered as the first initial study of its kind for *Schistocerca americana*, in terms of examining the changes in the color intensity of the chromatophores present in the wings caused by thermal transition under laboratory conditions. The color change can be considered as an indicator of a reaction to the increase or decrease of temperature ^2, 3^ as the clear surface (in this case the wings) reflects the radiation emitted by the lightbulbs and thus absorb less heat, while in the case of a decrease in temperature, the wings absorb more heat.

## Data availability

### Underlying data

Figshare: Microscopy photos of the wings. https://doi.org/10.6084/m9.figshare.7749404.v1 ^16^

Figshare: Raw data for Figure 2. https://doi.org/10.6084/m9.figshare.7749395.v1 ^13^

Data are available under the terms of the Creative Commons Attribution 4.0 International license (CC-BY 4.0).
