# Supplementary material for: Incidence of the thermal transition in the range of 45-5°C in chromatophores present in the wings of Schistocerca americana
Source: F1000Res. 2019 Feb 27;8:223. [Version 1] doi: 10.12688/f1000research.18314.1 (PMC6468705; doi:10.12688/f1000research.18314.1)
Supplement: Peer review history

# Peer review: Reviewer response for version 1

**DOI:** 10.5256/f1000research.20033.r47128

**Published:** 2019-04-15

**Authors:** Daniel A. Lowy

**Affiliations:** 1 VALOR HUNGARIAE Budapest, Budapest, Hungary; 2 NOVA University, Alexandria, VA, USA

The authors report their study on the variation of color intensity of the chromatophores present in the wings of *Schistocerca americana.* For this, 31 specimens were subjected to thermal transitions within a range of decreasing temperatures, from 45 to 5°C. Darkening and intensifying of color was revealed by optical microscopy of the insect wings.

 The reviewer believes that this study represents a novelty and is of interest to the scientific community. The authors' approach may have an impact in the monitoring of local climate changes. The manuscript is well written, has a good flow, and its English style is good. The observations listed below are intended to serve for further improving the quality of the paper.

**Observations regarding the content:**

1. Please, add to the Abstract, as well, the following statement of the Conclusion: "The color change can be considered as an indicator of a reaction to the increase or decrease of temperature as the clear surface (in this case the wings) reflects the radiation emitted by the light bulbs and, therefore, absorb less heat, while at decreasing temperature, the wings absorb the more and more heat." 
2. Please add to the Introduction: "It is believed that the detected color in natural environment may become an indicative of local climate change." The reviewer believes that instructing school students to survey the color of insects may represent a statistical means to reveal possible micro changes in climate.
3. Three additional references would be welcome: (i) at the end of section on Specimen collection: Reference (or web site) for the protocol of the Ministry of Environment of Ecuador; (ii) for iVinci Express v.4.6. (iii) for 0to225 software.
4. Housing of specimens – the second sentence is incomplete: "...the collection of specimens in the (???) and the others of greater longevity...etc." 

**Suggestions for style improvements:**

Introduction:

- The following sentence reads difficultly: “It is also known that chromatophores (to those) OF cells present in the animal integumentary system containing pigments, (and these cells) respond to hormonal factors and/or neuronal (response to stimuli) factors ^2^.” Please, delete the words in parentheses and add "OF".
- Last sentence of paragraph 1: "insects are at (an) advantage...because they ABSORB SOLAR LIGHT MORE EFFICIENTLY, SO THAT THEY warm up more quickly." Please, delete the words in parentheses and add the capitalized part for more accuracy.
- Paragraph 3: ...by the observation REPORTED in the literature (3-) etc.

Thermal transition:

- The temperature was set to 25°C  (rather than "was applied")...were exposed to DECREASING temperatures of 45°C, 40°C, etc.

Results and Discussions:

- ...at 45°C and 40°C  there are no (huge) SIGNIFICANT differences etc. At 35°C chromatophores of the wings become light brown, intensifyING their color (rather than "intensify"). 

I have read this submission. I believe that I have an appropriate level of expertise to confirm that it is of an acceptable scientific standard.

# Peer review: Reviewer response for version 1

**DOI:** 10.5256/f1000research.20033.r45936

**Published:** 2019-04-09

**Authors:** David Terán

**Affiliations:** 1 School of Chemistry and Molecular Biosciences, University of Queensland, Brisbane, Qld, Australia; 2 Universidad Técnica de Ambato, Ambato, Ecuador

**Incidence of the thermal transition in the range of 45-5°C in chromatophores present in the wings of *Schistocerca Americana***

 The manuscript presented by Cañizares and co-authors reports the effect of temperature (radiant heat) in wing chromatophores of *S. americana*. There are other publications about this topic, and the present manuscript is in accordance of reported results. However, the manuscript requires addressing of different issues to be accepted. The suggestions are the following:

**Introduction:**

- The citations 11-12 could have a peer review source.
- The description of the natural environment of *S. americana* is missing. This information will enrich the description of the hypothesis of the manuscript.  

 

**Material and methods:**

- The description of house-keeping is not clear.
- The range of temperatures is between 45-5˚C however there is a gap between 25-5˚C. 
- There is no statement about how the temperature was controlled.
- There is no statement of how long the *S. Americana* was kept at certain conditions. 

 

**Results and discussion:**

- It’s true that “Temperature, precipitation and solar radiation are the meteorological elements that most affect the distribution, rate of growth, reproduction, migration and adaptation of insects” as stated in paragraph 3 lines 1-3. However, are the authors sure that they can mention this in a very short time of exposure in their experiment? 
- The last paragraph about the negative correlation requires more discussion; as it is the most interesting part of the paper. 

I have read this submission. I believe that I have an appropriate level of expertise to confirm that it is of an acceptable scientific standard, however I have significant reservations, as outlined above.

# Peer review: Reviewer response for version 1

**DOI:** 10.5256/f1000research.20033.r45030

**Published:** 2019-03-14

**Authors:** Ana Danitza Peñafiel-Vinueza

**Affiliations:** 1 Laboratorio de Genética Evolutiva, Facultad de Ciencias Exactas y Naturales, Pontificia Universidad Católica del Ecuador, Quito, Ecuador

The presented research note addresses the effect of temperature on melanin pigment of wing chromatophores of *S. americana* by manipulating radiant heat. Despite the results found being in accordance with previous studies, there are a number of issues with the methods and results that need to be clarified. I present more specific comments, as follows:

**Introduction:**

- It would be helpful to have more information about the ecology and distribution of *S. americana*, to enrich the introduction and to correlate with the conclusions.

**Methods:**

- Housing of specimens: "first terrarium used for the collection of specimens in the (_) and the others…" – I think you wanted to state “the first terrarium used for the collection of specimens in the field”, if this is right and since the specimens were collected by hand, I understand that this first terrarium was used for carrying the specimens from the field to laboratory.
- Thermal transition: Not clear why, if thermal transition ranged from 45° to 5°C, the temperatures tested did not include the ranges from 20° to 10° and instead went directly from 25° to 5°C. Since the distribution of *S. americana* includes places where the temperature occurs in the range that is missing, testing these temperatures could complete the overview of thermal effects from the whole 45° to 5°C range. Otherwise, please clarify and state your reasons.
- If temperature is induced by manipulating radiant heat to reach the range 45° to 25°C, why did you use dry ice to induce the 5°C temperature? The methodology used in the laboratory should be homogeneous, otherwise it can increase variability in results.
- Data collection: How many wings were analyzed for each treatment? The number should be stated here, it seems that only one wing was analyzed for each treatment.

**Results and Discussions:**

- The statement “the majority of the studies that examine the thermal melanism have focused on species with very distinct color morphs representing a wide range in melanism” (third paragraph, line 7-9). Is taken almost exactly as is stated by Harris *et al.* (2013 ^1^) (reference #3 in your manuscript) in their conclusions.
- A discussion about how the negative correlation between temperature and pigmentation that you have found, relates with the adaptation to the environment where *S. americana* lives.

I have read this submission. I believe that I have an appropriate level of expertise to confirm that it is of an acceptable scientific standard, however I have significant reservations, as outlined above.

## References

[1] HarrisRMMcQuillanPHughesL: A test of the thermal melanism hypothesis in the wingless grasshopper Phaulacridium vittatum. *J Insect Sci*.2013;13: 10.1673/031.013.5101 51 10.1673/031.013.5101 23909454PMC3740933
